# DeepGOWeb: fast and accurate protein function prediction on the (Semantic) Web

**DOI:** 10.1093/nar/gkab373

**Published:** 2021-05-21

**Authors:** Maxat Kulmanov, Fernando Zhapa-Camacho, Robert Hoehndorf

**Affiliations:** Computational Bioscience Research Center, Computer, Electrical and Mathematical Sciences & Engineering Division, King Abdullah University of Science and Technology, 4700 King Abdullah University of Science and Technology, Thuwal 23955-6900, Saudi Arabia; Computational Bioscience Research Center, Computer, Electrical and Mathematical Sciences & Engineering Division, King Abdullah University of Science and Technology, 4700 King Abdullah University of Science and Technology, Thuwal 23955-6900, Saudi Arabia; Computational Bioscience Research Center, Computer, Electrical and Mathematical Sciences & Engineering Division, King Abdullah University of Science and Technology, 4700 King Abdullah University of Science and Technology, Thuwal 23955-6900, Saudi Arabia

## Abstract

Understanding the functions of proteins is crucial to understand biological processes on a molecular level. Many more protein sequences are available than can be investigated experimentally. DeepGOPlus is a protein function prediction method based on deep learning and sequence similarity. DeepGOWeb makes the prediction model available through a website, an API, and through the SPARQL query language for interoperability with databases that rely on Semantic Web technologies. DeepGOWeb provides accurate and fast predictions and ensures that predicted functions are consistent with the Gene Ontology; it can provide predictions for any protein and any function in Gene Ontology. DeepGOWeb is freely available at https://deepgo.cbrc.kaust.edu.sa/.

## Graphical Abstract

**Graphical Abstract:**
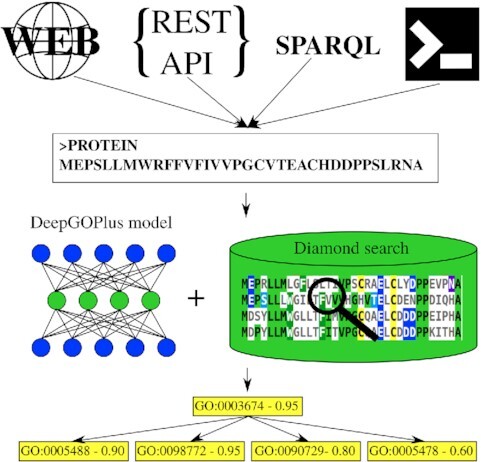
Overview of DeepGOWeb workflow. DeepGOWeb can be accessed through website, a REST API SPARQL and is available as a command line tool. It outputs predicted functions for a protein amino acid sequence and a confidence score for each function.

## INTRODUCTION

Many more protein sequences are known than can experimentally be investigated. Advances in sequencing technologies and applications of these technologies to areas such as metagenomics increase the amount of available protein sequences further. Understanding the functions of proteins is crucial to understanding the biological processes within organisms on a molecular level.

Several computational approaches to predicting protein functions have been developed (1). These approaches rely on different types of information that can be used to predict protein functions, including the protein sequence (2,3), interaction networks (4), gene expression (5), sequence similarity (6), phenotypes resulting from loss of function mutations (7) and text mining (8). The different types of information can often provide complementary information and, consequently, combining multiple types of information can often improve predictive performance (1). However, the only type of information that is available for the majority of sequenced proteins is the protein amino acid sequence (and, derived from this sequence, the sequence similarity to other proteins), whereas the position in interaction networks, text mining or gene expression data can only be obtained for some proteins. Therefore, while many features may improve predictive performance, they also limit the scope of function prediction.

Computational methods that predict function from sequence have to face two challenges; first, they need to find a way to extract or learn features from the protein sequence that are predictive of functions; and second, they have to ensure that predictions are consistent with biological background knowledge about functions and their interrelations. The first challenge is now commonly addressed through deep learning methods which learn ‘representations’ of protein sequences that can be used to predict functions (9). The second challenge relates to how predictions can be made consistent with the Gene Ontology (GO) (10) which is the structured vocabulary used to characterize protein functions and cellular locations, and contains over 40 000 different classes. Function prediction methods that are consistent with the GO rely either on structured, hierarchical classification methods that include the GO as background knowledge within the model itself, or they rely on post-processing where the GO is ignored within the model and predictions are post-processed to ensure consistency.

**Figure 1.**
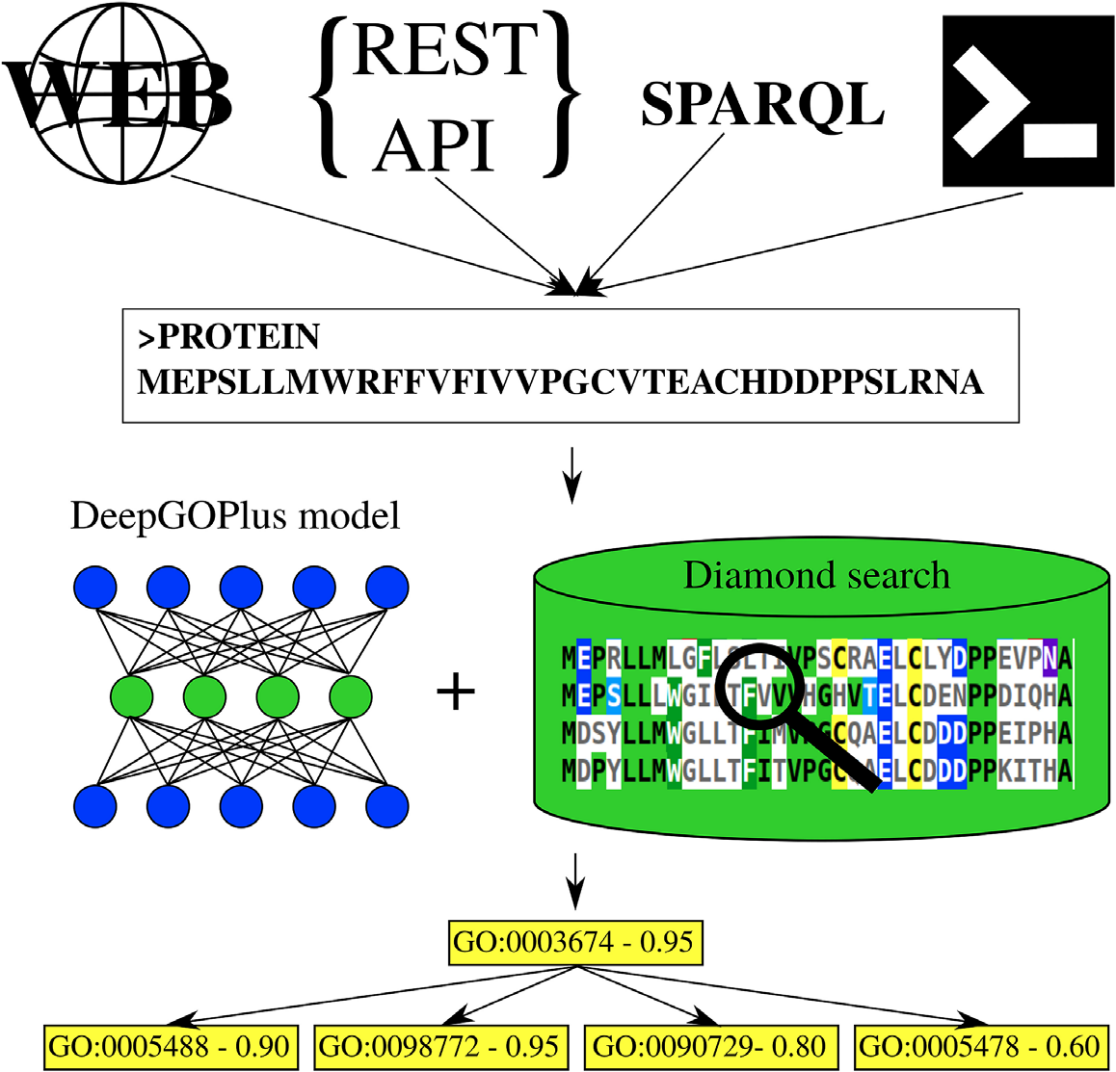
Overview of DeepGOWeb workflow. DeepGOWeb can be accessed through a website, a REST API, and SPARQL, and DeepGOPlus is available as a command line tool. The outputs of DeepGOWeb are the predicted functions for a protein amino acid sequence and a confidence score for each function.

DeepGOPlus (11) predicts protein functions through a combination of deep learning and sequence similarity to proteins with known functions, and ensures that predicted protein functions are consistent with the GO. When evaluating DeepGOPlus on the dataset used by the Critical Assessment of Function Annotation (CAFA) 3 challenge (1), DeepGOPlus achieves performance close to the state-of-the-art function prediction methods. DeepGOPlus is available as a standalone software, and DeepGOWeb makes the protein function prediction method DeepGOPlus available as a service. DeepGOWeb can be accessed through a website, a REST API and a SPARQL endpoint to interoperate with databases that rely on Semantic Web technologies (Figure 1).

## METHODS AND IMPLEMENTATION

### Materials and data

For training DeepGOPlus, we use reviewed and manually annotated protein sequences that are available in UniProtKB/Swiss-Prot (12) with their experimental Gene Ontology (GO) (10) function annotations. The experimental annotations are filtered using evidence codes EXP, IDA, IPI, IMP, IGI, IEP, TAS, IC, HTP, HDA, HMP, HGI and HEP. We update and retrain the model with every new release of UniProtKB.

### DeepGOPlus prediction model

**Figure 2.**
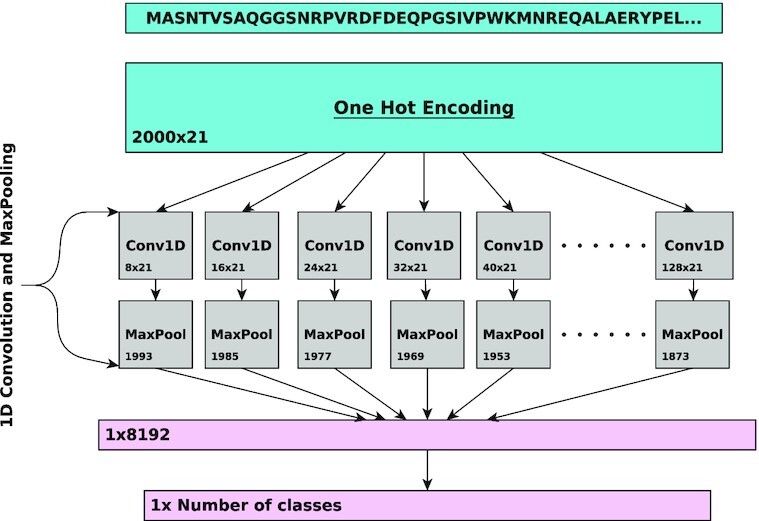
Overview of the CNN in DeepGOPlus. The CNN uses multiple filters of variable size to detect the presence of sequence motifs in the input amino acid sequence.

DeepGOPlus predicts protein functions based on the combination of Convolutional Neural Network (CNN) and sequence similarity methods (11). First, we train the CNN model to predict more than 5,000 GO classes that were annotated to at least 50 proteins based on experimental evidence. The CNN model consists of 16 1D-convolutional layers of sizes {8, 16, 24, ..., 128} with 512 filters in each layer. The convolutional layers are followed by a max-pooling layer which returns a value that determine whether a filter was active or not. The outputs are concatenated and passed to a fully connected layer with a sigmoid activation function for classification. Figure 2 depicts the architecture of the CNN model.

Second, for a query sequence we find sets of similar sequences from a training set using Diamond (13) with an *e*-value of 0.001 and obtain a bitscore for every similar sequence. We use GO class annotations of the similar sequences to annotate the query sequence. For a set of similar sequences *E* of the query sequence *q*, we compute the prediction score for a GO class *f* as

$$\begin{equation*} S(q, f) = \frac{\sum _{s \in E}^{} I(f \in T_{s}) \cdot bitscore(q, s)}{\sum _{s \in E}^{} bitscore(q, s)}\ , \end{equation*}$$

where *T*_*s*_ is a set of true annotations of the protein with sequence *s*. Then, to compute the final prediction scores of DeepGOPlus, we combine the two prediction scores using a weighted sum model (14):

$$\begin{equation*} S = \alpha \cdot S_{\text{DiamondScore}} + (1 - \alpha ) \cdot S_{\text{DeepGOCNN}} \ , \end{equation*}$$

where 0 ≤ α ≤ 1 is a weight parameter which balances the relative importance of the two prediction methods.

### Evaluation measures

We evaluate DeepGOPlus using standard CAFA evaluation metrics such as *F*_max _, *S*_min _ (15) and the area under the precision-recall curve (AUPR). We report the performance of every new release on the changelog of the DeepGOWeb website.

*F*_max _ is a maximum protein-centric F-measure computed over all prediction thresholds. First, we compute average precision and recall using the following formulas:

$$\begin{equation*} pr_{i}(t) = \frac{\sum _{f} I(f \in P_i(t) \wedge f \in T_i)}{\sum _{f} I(f \in P_i(t))} \end{equation*}$$

$$\begin{equation*} rc_{i}(t) = \frac{\sum _{f} I(f \in P_i(t) \wedge f \in T_i)}{\sum _{f} I(f \in T_i)} \end{equation*}$$

$$\begin{equation*} AvgPr(t) = \frac{1}{m(t)} \cdot \sum _{i=1}^{m(t)}pr_{i}(t) \end{equation*}$$

$$\begin{equation*} AvgRc(t) = \frac{1}{n} \cdot \sum _{i=1}^{n}rc_{i}(t) \end{equation*}$$

where *f* is a GO class, *T*_*i*_ is a set of true annotations, *P*_*i*_(*t*) is a set of predicted annotations for a protein *i* and threshold *t*, *m*(*t*) is a number of proteins for which we predict at least one class, *n* is a total number of proteins and *I* is an identity function which returns 1 if the condition is true and 0 otherwise. Then, we compute the *F*_max _ for prediction thresholds *t* ∈ [0, 1] with a step size of 0.01. We count a class as a prediction if its prediction score is higher than *t*:

$$\begin{equation*} F_{\max } = \max _{t}\left\lbrace \frac{2 \cdot AvgPr(t) \cdot AvgRc(t)}{AvgPr(t) + AvgRc(t)}\right\rbrace \end{equation*}$$

*S*_min _ computes the semantic distance between real and predicted annotations based on information content of the classes. The information content *IC*(*c*) is computed based on the annotation probability of the class *c*:

$$\begin{equation*} IC(c) = -log(Pr(c|P(c)) \end{equation*}$$

where *P*(*c*) is a set of parent classes of the class *c*. The *S*_min _ is computed using the following formulas:

$$\begin{equation*} S_{\min } = \min _{t}\sqrt{ru(t)^2 + mi(t)^2} \end{equation*}$$

where *ru*(*t*) is the average remaining uncertainty and *mi*(*t*) is average misinformation:

$$\begin{equation*} ru(t) = \frac{1}{n}\sum _{i=1}^{n}\sum _{c \in T_i - P_i(t)}IC(c) \end{equation*}$$

$$\begin{equation*} mi(t) = \frac{1}{n}\sum _{i=1}^{n}\sum _{c \in P_i(t) - T_i}IC(c) \end{equation*}$$

### Implementation

DeepGOPlus is implemented using the TensorFlow (16) library and trained on Nvidia Titan X and P6000 GPUs with 12–24 Gb of RAM. In order to tune the different parameters of the convolutional neural network model and its architecture, we performed an extensive search and selected the best model based on a validation set performance. The DeepGOWeb application and REST API is implemented using Django Framework (https://www.djangoproject.com/) with the Django REST Framework (https://www.django-rest-framework.org/).

The SPARQL endpoint is implemented using the Apache Jena ARQ query engine. The endpoint uses custom functions which can be called within a SPARQL query. The endpoint uses the REST API to obtain predictions.

## RESULTS

### DeepGOWeb predictions and access

DeepGOWeb is a webserver that takes a set of protein sequences as an input and outputs the predicted functions of the proteins. Protein sequences can be provided in FASTA format or as strings separated by new lines. An additional threshold parameter can be used to select the minimum confidence in function predictions. The default value of the prediction threshold parameter is 0.3 which results in the best performance of DeepGOPlus using the *F*_max _ measure. Lowering the threshold parameter may help to obtain more specific annotations; however, it may also result in more incorrect predictions.

For each protein in a request to DeepGOWeb, the prediction results consist of a list of pairs; each pair consists of a GO class and a confidence score. Table 1 shows an example prediction for the zebrafish protein PP2A subunit B isoform delta (UniProt:Q6NY64).

**Table 1.** Example predictions for the zebrafish protein PP2A subunit B isoform delta (UniProt:Q6NY64). We only show predictions for the *Cellular Component* branch of GO; the DeepGOWeb output will also include a similar list of predictions for *Molecular Function* and *Biological Process*. The prediction confidence threshold is the default of 0.3. Predictions of DeepGOPlus are consistent with the GO and confidence scores monotonically decrease from a class to its subclasses. As a result, the root class within each of the branches of GO (*Cellular Component* in this example) will always have the highest confidence score

Cellular component		
GO:0110165	Cellular anatomical entity	0.782
GO:0005622	Intracellular anatomical structure	0.780
GO:0043226	Organelle	0.689
GO:0043229	Intracellular organelle	0.689
GO:0005634	Nucleus	0.600
GO:0043227	Membrane-bounded organelle	0.600
GO:0043231	Intracellular membrane-bounded organelle	0.600
GO:0032991	Protein-containing complex	0.507
GO:0005737	Cytoplasm	0.444
GO:1902494	Catalytic complex	0.364
GO:0005829	Cytosol	0.305

The true path rule (10) in the GO requires that, if *C* is a subclass of *D*, then any protein with function *C* will also have the function *D*. The prediction model of DeepGOPlus does not directly enforce the consistency of predictions, and it is possible that the predicted functions are inconsistent with the true path rule in GO. Consistency is enforced by DeepGOPlus in a post-processing step to ensure that, for any class *D*, the confidence of the prediction of *D* (for any protein) is the maximum of the prediction of *D* and the confidence for the predictions of any subclass of *D*. DeepGOWeb only outputs the processed predictions that are consistent with the true path rule in GO. As a result, the confidence score of predictions monotonically decreases with the depth in the GO hierarchy (see Table 1).

DeepGOPlus combines a deep learning model with similarity-based predictions. Given a query protein, similar proteins with known functions are identified using sequence similarity and their GO annotations are combined with the predictions of the deep learning model. The proteins that were used to obtain similarity-based predictions are returned by DeepGOPlus as well together with their similarity score (bitscore) to the query protein; these proteins can be explored to identify the origins and provenance of similarity-based predictions.

We provide four different ways for accessing DeepGOPlus. DeepGOPlus can be installed as a command line tool. Installation can be either from the main git repository, using the Python pip package manager, or using a Docker container. The command line tool for DeepGOPlus is suitable for installation on single machines, compute clusters, or as part of (containerized) computational workflows.

DeepGOWeb is a website that makes DeepGOPlus predictions available through a web-based user interface. The website allows users to specify protein sequences and the confidence threshold and explore the DeepGOPlus predictions. The output of a prediction consists of a sorted list of GO function predictions; the list is separated by the GO sub-hierarchy (molecular function, biological process, cellular component) and sorted by prediction confidence. Additionally, the website allows exploring the proteins that were used for similarity-based predictions; this list of proteins is sorted by the similarity to the query protein, and each protein is linked to its entry in the UniProt database (12). Prediction results can be downloaded in JSON format together with the confidence scores. The DeepGOWeb website limits the amount of proteins for which functions can be predicted in a single query to 10 to ensure an adequate response time.

DeepGOWeb can also be accessed through a REST API; the API allows access to DeepGOPlus from software applications without installing the command line tool, where computational resources are not sufficient to run DeepGOPlus locally, or when including DeepGOPlus predictions as part of workflows. We limit the amount of sequences that can be submitted through the API in a single request to 100; the webserver processes a single request in one thread and the limit of 100 sequences limits the runtime of this thread and ensures that new requests are treated fairly when queued.

Finally, DeepGOWeb provides a SPARQL endpoint to access DeepGOPlus predictions. SPARQL (17) is a query language for data in the Resource Description Framework (RDF) (18) and use in the Semantic Web (19). Many databases in the life sciences now make their data available through public SPARQL endpoints (20); in particular UniProt (12) provides one of the longest-running SPARQL endpoints for access to its data. Use of DeepGOPlus through its SPARQL endpoint provides interoperability with this growing set of Semantic Web resources in the life sciences. For example, we can query proteins in UniProt using their SPARQL endpoint and calling DeepGOPlus within a single query:

PREFIX dg: <http://deepgoplus.bio2vec.net/functions#>

PREFIX GO: <http://purl.obolibrary.org/obo/GO_>

PREFIX rdf: <http://www.w3.org/1999/02/22-rdf-syntax-ns#>

PREFIX up: <http://purl.uniprot.org/core/>

PREFIX uniprot: <http://purl.uniprot.org/uniprot/>

SELECT ?sub ?go ?label ?score

WHERE

{

{

SELECT ?aa_sequence

WHERE

{

SERVICE <http://sparql.uniprot.org/sparql> {

uniprot:Q6NY64 up:sequence ?isoform .

?isoform rdf:value ?aa_sequence .

}

}

}

(?sub ?go ?label ?score) dg:deepgo (?aa_sequence 0.3) .

}

This query will retrieve the sequence of the zebrafish protein PP2A subunit B isoform delta (UniProt:Q6NY64) from the UniProt SPARQL endpoint and return the DeepGOWeb function predictions with a threshold of 0.3 for this protein.

### Updates and versioning

DeepGOPlus outputs protein functions using the Gene Ontology (GO) (10) and is trained on the curated version of UniProt called Swiss-Prot (12). The GO changes regularly by adding and removing classes, and Swiss-Prot keeps expanding and adding new curated information about proteins and their functions. It is therefore important for DeepGOPlus to be updated regularly to reflect these changes to training data as well as the functions used as output.

For DeepGOWeb, we have implemented an automated process that periodically checks for a new release of the Swiss-Prot data and retrains the model accordingly. Each time new training data becomes available, the GO is also updated to reflect any added or removed classes. After training, the new DeepGOPlus model is released with a new version number and release data and the DeepGOWeb website updated. For each release of DeepGOPlus, we compute evaluation metrics and include them in the release notes as well as in the DeepGOWeb website as a record of the evolution of the model performance.

To ensure reproducibility, every release of DeepGOPlus is archived and contains the trained model, data files, evaluation scores, and all the necessary files to reproduce the results shown in the release notes. This data can be accessed at https://deepgo.cbrc.kaust.edu.sa/data/. Each release is named after its version and the versioning format we follow has the form a.b.c where c is the number updated when a new model is released using new Swiss-Prot data. For prediction, the old models can be used to reproduce results that were obtained with a specific version of DeepGOPlus. DeepGOWeb and the DeepGOPlus command line prediction tool all take an optional parameter to specify the version of DeepGOPlus to use; the default is always to use the latest version of DeepGOPlus.

### Benchmarking and comparison

**Figure 3.**
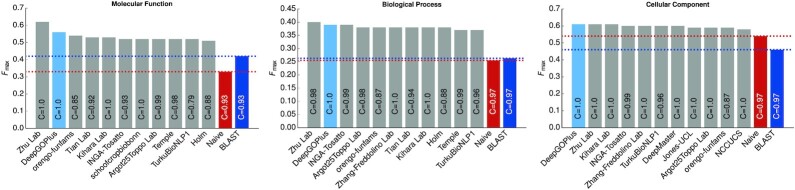
Comparison of DeepGOPlus with CAFA3 top 10 methods.

We continuously evaluate DeepGOPlus using the evaluation methods of the Critical Assessment of Function Annotation (CAFA) (21) challenge. For comparison with other prediction methods, we use the CAFA3 challenge data (1) and evaluation method. We generated a time-based split of training and testing datasets. The training set contains all proteins with experimental annotations available before February 2017, and the testing set includes newly annotated proteins between February 2017 and November 2017. We compared DeepGOPlus with the top performing methods in CAFA3 using the *F*_max _ evaluation metric. DeepGOPlus resulted in the highest *F*_max _ in the Cellular Component (CC) subontology evaluation and the second best performance in Biological Process (BP) and Molecular Function (MF) subontology evaluations. Figure 3 shows the comparison of DeepGOPlus with all CAFA3 top performing methods.

The newer versions of DeepGOPlus obtain higher *F*_max_; for example, version 1.0.3 has an *F*_max_ of 0.647, 0.531 and 0.685 for MF, BP and CC, respectively. However, these results cannot easily be compared with other methods as the training and testing data, as well as the GO ontology, are no longer identical to the data used by other methods. We will continue to update the DeepGOWeb website with the performance of DeepGOPlus in official CAFA challenges as they become available.

To ensure practical utility of DeepGOPlus for prediction functions for a large number of protein sequences, we also evaluated the processing time both of DeepGOPlus directly and of the DeepGOWeb webserver. When using an Nvidia Titan X GPU for processing, the DeepGOPlus command line tool can predict functions for around 40 sequences per second. The REST API can process ∼5 protein sequences per second when using batches of 100 sequences; the DeepGOWeb website is the slowest way to access DeepGOPlus and requires on average 13 seconds to predict functions for 10 protein sequences.

We compare DeepGOWeb with several other function prediction web servers such as SIFTER (22), PredictProtein (23), ECPred (24), NETGO (4), CATH/Gene3D (25), ProFunc (26), InterProScan (27), I-TASSER (28), PANDA (29), DEPICTER (30) and FFPRED3 (31) in terms of their functionality and accessibility. These servers differ from DeepGOWeb either in that they limit the organisms for which protein functions are predicted; do not predict functions using the GO but other functional categories, or only use parts of the GO; limit the type of proteins for which functions are predicted; do not have a predictive performance comparable to DeepGOPlus; or require substantially more time for predicting functions of one protein. To our knowledge, no function prediction server is available through SPARQL. Table 2 provides a comparison of function prediction web servers.

**Table 2.** Comparison of different function prediction web servers and DeepGOWeb

	Open source	REST API	Command line	SPARQL	Immediate results	Sequence only	Predict GO classes	3D Structure based	Any species
**DeepGOWeb**	✓	✓	✓	✓	✓	✓	✓		✓
SIFTER	✓		✓			✓	✓		✓
PredictProtein	✓		✓			✓	✓		✓
ECPred	✓		✓			✓			✓
NETGO							✓		✓
CATH/Gene3D	✓	✓	✓		✓	✓		✓	✓
FFPRED3	✓		✓			✓	✓		
ProFunc								✓	✓
InterProScan	✓	✓	✓		✓	✓			✓
PANDA						✓	✓		✓
I-TASSER			✓			✓		✓	✓
DEPICTER						✓			✓

## CONCLUSIONS

DeepGOWeb is a webserver for obtaining fast and accurate functional annotations for proteins. DeepGOPlus implements a function prediction method that relies only on protein sequences and focuses on providing predictions quickly. These design decisions allow DeepGOPlus and DeepGOWeb to be applied to a wide range of use cases. In particular, DeepGOWeb can be used to provide whole genome functional annotations of newly sequenced organisms for which no additional information is available. DeepGOPlus has previously been used to annotate newly sequenced crop plants, in particular fonio millet (*Digitaria exilis*) (32), and early SARS-CoV-2 sequences (33); however, DeepGOPlus places no restrictions on the protein sequences and can be used to prediction functions for proteins from any organism. DeepGOPlus and DeepGOWeb are available as Free Software (34) at https://deepgo.cbrc.kaust.edu.sa/.
